# Effects of lifestyle on hepatobiliary enzyme abnormalities following the Fukushima Daiichi nuclear power plant accident: The Fukushima health management survey

**DOI:** 10.1097/MD.0000000000012890

**Published:** 2018-10-19

**Authors:** Atsushi Takahashi, Tetsuya Ohira, Kanako Okazaki, Seiji Yasumura, Akira Sakai, Masaharu Maeda, Hirooki Yabe, Mitsuaki Hosoya, Akira Ohtsuru, Yukihiko Kawasaki, Hitoshi Suzuki, Michio Shimabukuro, Yoshihiro Sugiura, Hiroaki Shishido, Yoshimitsu Hayashi, Hironori Nakano, Gen Kobashi, Kenji Kamiya, Hiromasa Ohira

**Affiliations:** a Department of Gastroenterology, Fukushima Medical University School of Medicine; b Radiation Medical Science Center for the Fukushima Health Management Survey; c Department of Epidemiology; d Department of Public Health; e Department of Radiation Life Sciences; f Department of Disaster Psychiatry; g Department of Neuropsychiatry; h Department of Pediatrics; i Department of Radiation Health Management; j Department of Cardiology; k Department of Diabetes, Endocrinology and Metabolism; l Department of Neurology; m Department of Orthopaedic Surgery; n Department of Nephrology and Hypertension, Fukushima Medical University School of Medicine, Fukushima; o Department of Public Health, Dokkyo Medical University School of Medicine, Shimotsuga, Japan.

**Keywords:** disaster-related factors, Fukushima Daiichi, nuclear power plant accident, hepatobiliary enzyme abnormality, lifestyle factors

## Abstract

Dramatic lifestyle changes due to the Fukushima Daiichi Nuclear Power Plant accident increased the prevalence of hepatobiliary enzyme abnormalities (HEA). We aimed to evaluate associations of HEA with specific lifestyle- and disaster-related factors in residents who lived near the Fukushima Daiichi Nuclear Power Plant.

This cross-sectional study included 22,246 residents who underwent a Comprehensive Health Check and the Mental Health and Lifestyle Survey from June 2011 to March 2012. Residents were divided into 2 groups based on residential area and housing status after the accident. Associations between HEA and lifestyle- and disaster-related factors, including psychological distress, were estimated using logistic regression analysis adjusted for demographic and lifestyle factors.

HEA was present in 27.3% of subjects. The prevalence of HEA was significantly higher in evacuees than controls (29.5% vs 25.7%, *P* < .001). There were significant differences in various lifestyle characteristics and the prevalence of post-traumatic stress disorder between evacuees and controls. Multivariable logistic regression analysis showed that age, sex, moderate to heavy drinking, and low/no physical activity were significantly associated with HEA regardless of evacuation status. Changes in jobs and unemployment were significantly associated with HEA in controls and evacuees, respectively.

Lifestyle and disaster-related factors, but not psychological distress, were associated with HEA among subjects who lived near the Fukushima Daiichi Nuclear Power Plant accident.

## 1 Introduction

Disasters change daily lifestyle factors and influence the health of affected residents. The Great East Japan Earthquake and the associated tsunami and accident at the Fukushima Daiichi Nuclear Power Plant that occurred in March 2011 forced more than 1,60,000 residents of Fukushima Prefecture to evacuate the area. The Fukushima Health Management Survey (FHMS) was created to monitor the long-term health of residents of this area following the accident.^[1]^ This FHMS is composed of four major elements: the Comprehensive Health Check, the Mental Health and Lifestyle Survey, thyroid ultrasonography, and a survey of pregnant women and nursing mothers.

We previously identified an increase in the prevalence of hepatobiliary enzyme abnormality (HEA) following the accident,^[2,3]^ as well as many other diseases included in the Comprehensive Health Check of the FHMS.^[2,4–10]^ In addition, the mental health and lifestyle survey of the FHMS showed that psychological distress following the accident was associated with changes in dietary and alcohol intake, sleep satisfaction, and a reduction of physical activity.^[11–16]^

Lifestyle changes affect HEA, but associations between specific lifestyle factors or mental health status and HEA following the accident have never been investigated. The aim of the present study was to elucidate factors associated with HEA following the Fukushima Daiichi Nuclear Power Plant accident based on results of the Comprehensive Health Check and the Mental Health and Lifestyle Survey.

## 2 Methods

### 2.1 Study population

Subjects were residents of evacuation-designated areas near the Fukushima Daiichi Nuclear Power Plant. They were evacuated from all areas of Hirono-machi, Naraha-machi, Tomioka-machi, Kawauchi-mura, Okuma-machi, Futaba-machi, Namie-machi, Katsurao-mura, Iitate-mura, and part of Tamura City, Minamisoma City, Kawamata-machi, and Date City.^[2]^ In 2010, the target population of these communities included 91,554 people. From June 2011 through March 2012, 37,058 people (15,519 men and 21,539 women) from these communities participated in both the Comprehensive Health Check and the Mental Health and Lifestyle Survey. The rate of participation for the target population was 40.5%. We excluded 14,812 participants due to insufficient data on HEA, lifestyle- and disaster-related factors, or psychological distress. Ultimately, 22,246 participants (10,936 men and 11,310 women) were eligible for our analyses.

The Ethics Committee of Fukushima Medical University approved this study (#29064). Informed consent was obtained from community representatives to conduct an epidemiological study based on guidelines of the Council for International Organizations of Medical Science. All participants in the FHMS provided written informed consent.

### 2.2 Definitions and data collection

Height and weight were measured (without shoes and in light clothing), and body mass index (BMI) was calculated as weight (kg)/height (m)^2^. Overweight and obesity were defined as BMI ≥ 25 kg/m^2^ and BMI ≥ 30 kg/m^2^, respectively. Systolic and diastolic blood pressures (SBP/DBP) were measured by trained technicians. Hypertension was defined as SBP ≥ 140 mmHg, DBP ≥ 90 mmHg, or the use of antihypertensive agents.

The following laboratory data were obtained: aspartate aminotransferase (AST; U/L); alanine aminotransferase (ALT; U/L); gamma-glutamyl transferase (γ-GTP; U/L); high-density lipoprotein cholesterol (mg/dL); low-density lipoprotein cholesterol (mg/dL); triglycerides (mg/dL); fasting plasma glucose (mg/dL); and hemoglobin A1c (% of total hemoglobin). We defined HEA as follows, based on the definition of the Ministry of Health, Labor, and Welfare: AST ≥ 31 U/L or ALT ≥ 31 U/L or γ-GTP ≥ 51 U/L.^[17]^ Diabetes and dyslipidemia were defined as previously reported.^[2]^ Subjects were divided into 2 groups by residential area and housing status. Evacuees included 9541 participants who were living in evacuated areas, including Hirono-machi, Naraha-machi, Tomioka-machi, Kawauchi-mura, Okuma-machi, Futaba-machi, Namie-machi, Katsurao-mura, and Iitate-mura and who moved into shelters or temporary housing in Tamura City, Minamisoma City, Kawamata-machi, and Date City. Controls included 12,705 participants who did not live in shelters or temporary housing in Tamura City, Minamisoma City, Kawamata-machi, and Date City.

In the Mental Health and Lifestyle Survey, the Japanese versions of the Kessler 6-item scale (K6)^[18]^ and Post-traumatic Stress Disorder Checklist (PCL-S)^[19]^were used to assess the status of participants’ mental health. The K6 consists of 6 brief questions about depressive and anxiety symptoms during the past 30 days, with overall scores ranging from 0 to 24. We defined psychological distress as corresponding to a K6 score of ≥ 13.^[20]^ The PCL-S is a tool to evaluate symptoms of post-traumatic stress disorder (PTSD) during the past 30 days. The PCL-S consists of 17 items, and the overall score ranges from 17 to 85. We classified participants as having probable PTSD if their overall PCL-S score was ≥ 44.^[19]^ In addition to the K6 and PCL-S, the questionnaires included medical history and various lifestyle factors, such as physical activity, sleep dissatisfaction, cigarette smoking, alcohol intake, and job status. Job status included unemployment or a change in job after the accident. Based on previous studies, alcohol intake was defined as non-drinker, light drinker (≤ 22 g of ethanol per day), or moderate/heavy drinker (>22 g of ethanol per day). ^[2,21]^

### 2.3 Statistical analysis

Evacuees and controls were compared using the *χ*^*2*^ test and Fisher exact test for categorical variables and the Mann–Whitney *U*test for continuous variables. We tested associations between HEA after the accident and other primary factors using simple and multivariable logistic regression analysis. The following variables were considered primary factors: age, sex, evacuation (yes or no), current smoking (yes or no), alcohol intake (non-drinker, light drinker, moderate/heavy drinker), sleep dissatisfaction (yes or no), physical activities (no, once a week, 2–4 times a week, every day), change of job (yes or no), unemployment (yes or no), psychological distress (yes or no), and PTSD (yes or no). SAS version 9.3 (SAS Institute, Cary, NC) was used for all statistical analyses. All probability values for statistical tests were 2-tailed, and *P* values < .01 were considered statistically significant.

## 3 Results

### 3.1 Characteristics of the study population

Overall, HEA was observed in 27.3% of the 22,246 participants. The prevalence of HEA was significantly higher in evacuees than controls (Table 1). Similarly, overweight and obesity were more prevalent in evacuees.

**Table 1:**
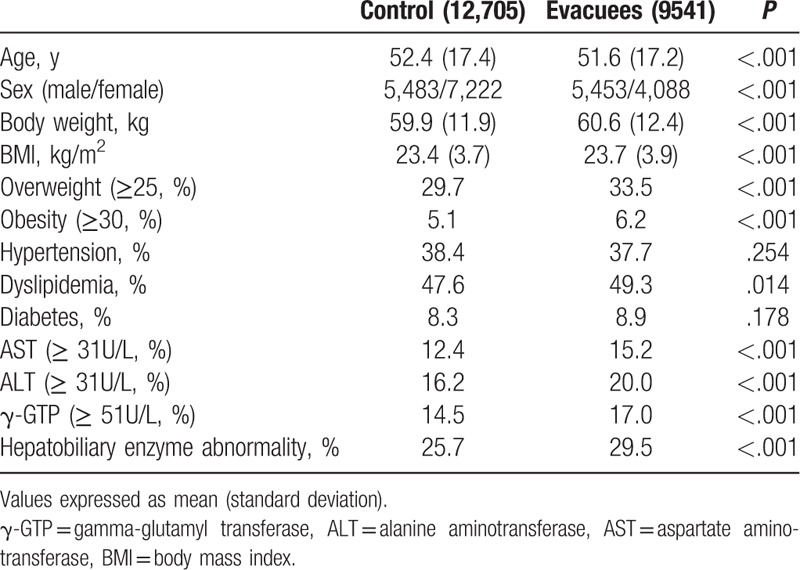
Clinical and biochemical characteristics of 22,246 participants.

### 3.2 Lifestyle characteristics

Lifestyle characteristics are shown in Table 2. There were significant differences in several lifestyle characteristics between evacuees and control. Significantly more evacuees changed their job or were unemployed compared with controls (66.1% vs 42.4%, *P* < .001 and 34.0% vs 12.6%, *P* < .001, respectively). More than half of participants engaged in no physical activity following the accident. Evacuees showed significantly (*P* < .001) higher levels than controls in terms of prevalence of psychological distress (16.4% vs 9.9%) and PTSD (22.9% vs 14.6%).

**Table 2:**
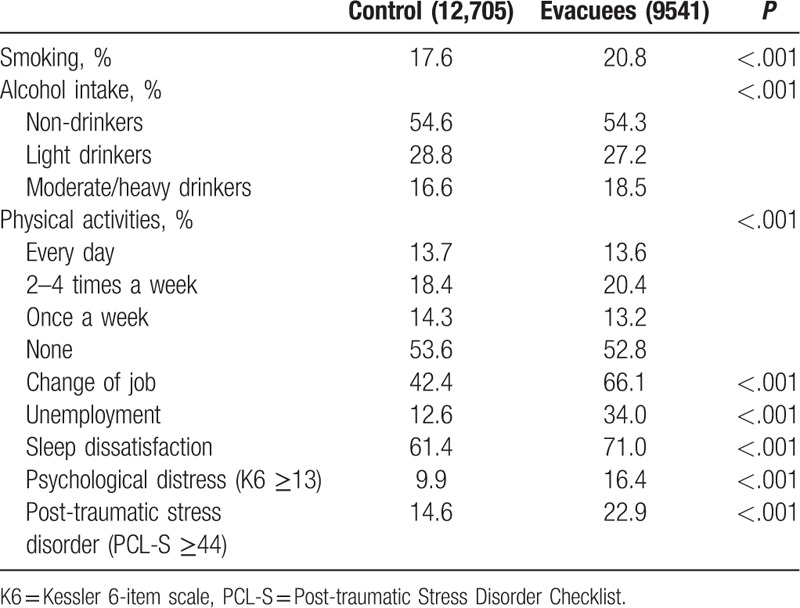
Lifestyle characteristics of 22,246 participants.

### 3.3 Lifestyle and disaster-related factors associated with HEA after the accident

In age- and sex-adjusted logistic analysis, smoking, moderate to heavy alcohol intake, lack of physical activity or physical activity once per week, and change of job were significantly associated with HEA in both groups (Table 3). Unemployment was significantly associated with HEA only in evacuees. Psychological distress and PTSD were not associated with HEA in either group.

**Table 3:**
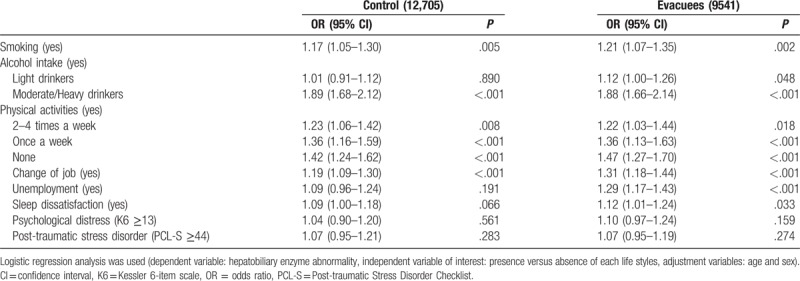
Age- and sex-adjusted logistic regression analysis of factors influencing hepatobiliary enzyme abnormality after the disaster among 22,246 participants.

Multivariable logistic regression was used to elucidate lifestyle and disaster-related factors associated with HEA after the accident (Table 4). In both groups, age, sex, moderate to heavy drinking, lack of physical activity, and physical activity once per week were significantly associated with HEA. Change of job was significantly associated with HEA in controls, whereas unemployment was significantly associated with HEA in evacuees.

**Table 4:**
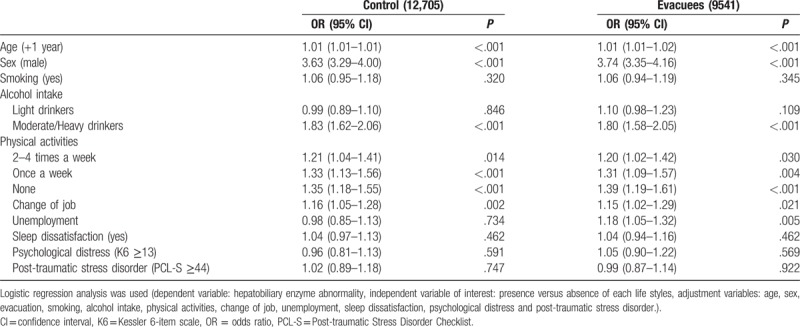
Multivariable logistic regression analysis of factors influencing hepatobiliary enzyme abnormality after the disaster among 22,246 participants.

## 4 Discussion

HEA increased after the Great East Japan Earthquake and subsequent Fukushima Daiichi Nuclear Power Plant accident.^[2,3]^ Based on the results of the Comprehensive Health Check of the FHMS, we recently reported that evacuation following the accident was an independent risk factor for HEA.^[2]^ In the present study, we showed the associations of lifestyle- and disaster-related factors with HEA after the accident based on the results of the Comprehensive Health Check and the Mental Health and Lifestyle Survey of FHMS.

Low levels or lack of physical activity or exercise has been reported to be associated with HEA.^[22–24]^ In our study, this association was confirmed among residents of a disaster-affected region. We also recently reported that improvements in daily physical activity reduced the incidence of HEA 3 to 4 years after the Great East Japan Earthquake and subsequent Fukushima Daiichi Nuclear Power Plant accident.^[21]^ As noted, this study confirmed the importance of physical activity as a factor influencing HEA, although it did not provide long-term data.

Following the Fukushima Daiichi Nuclear Power Plant accident, residents of the surrounding areas had to evacuate and change housing. In the present study, we redefined evacuees based on housing status in addition to residential area and confirmed that evacuation following the accident was an independent risk factor for HEA as previously reported.^[2]^ A previous study reported that after the Great East Japan Earthquake, prolonged living in temporary housing was significantly associated with increased alcohol intake and decreased exercise compared to owning a home.^[25]^ Moreover, housing type was associated with increases in γ-GTP levels after the Great East Japan Earthquake.^[26]^ Having to evacuate your home is thus an important factor that needs to be addressed appropriately to help protect against HEA following a disaster.

Change of job and unemployment were significantly associated with HEA in the present study. Job status can influence sleep dissatisfaction and physical activity,^[27,28]^ and unemployment leads to increased consumption of unhealthy foods such as snacks and fast foods.^[29]^ We recently reported that BMI was an independent factor influencing the incidence of HEA following the Great East Japan Earthquake,^[2]^ and previous studies have shown that unemployment is significantly associated with obesity.^[30,31]^ Change of job and unemployment could therefore be associated with HEA via the impact of these factors on obesity.

In this study, age- and sex-adjusted logistic regression analysis showed significant associations between HEA and sleep dissatisfaction. However, multivariable analysis demonstrated no significant association between sleep dissatisfaction and HEA.

Psychological distress after the Great East Japan Earthquake and subsequent Fukushima Daiichi Nuclear Power Plant accident has been a serious problem.^[32]^ Although we did not find a significant association between HEA and psychological distress or PTSD in the present cross-sectional study, FHMS has shown that psychological distress influenced many lifestyle factors such as physical activity, alcohol intake, diet, and sleep status.^[13–16]^ A longitudinal study may be able to clarify the association between HEA and these disaster-related factors.

The exposure levels of radiation in residents living around the Fukushima Nuclear Power Plant were very low because of early evacuation after the accident. The individual external doses of the residents in the first 4 months after the accident were ≤ 5 mSv in 97.4% of people,^[33]^ which is below the level of exposure during a single computed tomography scan. Indeed, when we further analyzed the association between external radiation doses and municipalities^[34]^ and prevalence of HEA, no significant associations were seen (r = 0.29, *P* = 0.34). Therefore, the causes of HEA are unlikely to be due to radiation exposure.

A particular strength of our study is our analyses of associations between HEA and detailed lifestyle factors, including mental status. A limitation of the study is that it was based on an analysis of routine health checkups in some residents, such that no definitive diagnoses of HEA were established. Therefore, it is impossible to deny the possibility of sample bias. Furthermore, because the response rate in the present study was relatively low, findings may not be generalizable to other populations. In addition, evaluation of food intake, which can affect obesity and HEA, will be an important element of future investigations. Drug-induced liver injury is a major cause of HEA. Residents who have sleep dissatisfaction and psychological distress can increase drug use and self-medication. In fact, previous reports showed increasing drug use for PTSD after various disasters.^[35–37]^ On the other hand, metabolic diseases have been shown to increase after a disaster.^[5,6,10]^ Drug treatment for metabolic diseases may also increase HEA. Therefore, drug use would be essential to elucidate the cause of HEA after the Fukushima Nuclear Power Plant accident in future studies. Moreover, we have no information about lifestyle before the Fukushima Daiichi Nuclear Power plant accident. A future longitudinal study of changes in both HEA and lifestyle factors will help clarify the true associations between HEA and lifestyle factors after the Fukushima Daiichi Nuclear Power Plant accident.

## 5 Conclusion

Several lifestyle- and disaster-related factors were associated with HEA after the Fukushima Daiichi Nuclear Power Plant accident. These findings could be important to lifestyle and living environment recommendations for individuals with HEA, regardless of evacuation status.

## Author contributions

All authors participated in study conception and design. Atsushi Takahashi, Tetsuya Ohira, and Kanako Okazaki performed statistical analysis of the data. Hiromasa Ohira supervised the project. All authors participated in interpretation of the results and drafting of the article, and approved the final version.

**Conceptualization:** Atsushi Takahashi, Tetsuya Ohira.

**Formal analysis:** Atsushi Takahashi, Tetsuya Ohira, Kanako Okazaki.

**Supervision:** Seiji Yasumura, Akira Sakai, Masaharu Maeda, Hirooki Yabe, Mitsuaki Hosoya, Akira Ohtsuru, Yukihiko Kawasaki, Hitoshi Suzuki, Michio Shimabukuro, Yoshihiro Sugiura, Hiroaki Shishido, Yoshimitsu Hayashi, Hironori Nakano, Gen Kobashi, Kenji Kamiya, Hiromasa Ohira.

**Writing – original draft:** Atsushi Takahashi.

**Writing – review & editing:** Atsushi Takahashi, Tetsuya Ohira.

## Acknowledgments

We thank the expert committee members, advisors, and staff of the Fukushima Health Survey Group for conducting this survey and for their support. This work was supported by the National Health Fund for Children and Adults Affected by the Nuclear Incident. The findings and conclusions of this article are solely the responsibility of the authors and do not represent the official views of the Fukushima Prefectural Government.
